# *Listeria monocytogenes*: possible mechanism of infection of goat uterus and its effects on uterine autophagy and cell apoptosis

**DOI:** 10.3389/fvets.2024.1413523

**Published:** 2024-08-15

**Authors:** Hailong Hong, Yunhai Hu, Siyuan Shi, Ben Liu, Wenya Zheng, Ruonan Bo, Zhongjie Xu, Yifan Wu, Yu Cao

**Affiliations:** ^1^College of Life Science and Resources and Environment, Yichun University, Yichun, China; ^2^Jiangxi Lvke Agriculture and Animal Husbandry Technology Co., Ltd., Yichun, China; ^3^College of Veterinary Medicine, Jiangsu Co-innovation Center for Prevention and Control of Important Animal Infectious Diseases and Zoonoses, Yangzhou University, Yangzhou, China

**Keywords:** *Listeria monocytogenes*, goat, listeriolysin O, E-cadherin, c-Met, apoptosis, autophagy

## Abstract

Listeriosis is highly prevalent in the animal farming industry, with *Listeria monocytogenes* as the causative pathogen. To identify potential therapeutic targets for LM infection, we investigated the mechanisms of LM infection in goat uteri. We inoculated a group of goats with LM via jugular vein injection, isolated and raised them, and subsequently collected sterile samples of their uterine tissue after they exhibited clinical symptoms of LM infection. We used Giemsa staining, immunohistochemical staining, real-time qPCR, and Western blotting as experimental methods.First, we investigated the mechanism of Listeria monocytogenes (LM) infection in the goat uterus by examining the expression levels of listeriolysin O, E-cadherin, and tyrosine kinase c-Met in the uterus.Furthermore, we investigated the impact of LM infection on uterine autophagy and cell apoptosis. The results indicate that the injection of LM into the goats’ jugular veins leads to LM infection in the goats’ uteri. During LM survival inside the goat uterine cells, there is a significant increase in the expression levels of LLO, E-cadherin, and c-Met in the host uterine tissue. This suggests that LM may potentially infect goat uteri through the InlA/E-cadherin and InlB/c-Met pathways. Furthermore, LM infection increases the levels of apoptosis and autophagy in goat uteri. Apoptosis genes Bcl-2 and Bax, as well as autophagy-related genes LC3B, PINK1, and Parkin, exhibit varying degrees of changes in localization and expression in goat uteri, mediating the occurrence of apoptotic and autophagic responses.

## 1 Introduction

*Listeria monocytogenes*, a Gram-positive bacterium described in 1926, displays remarkable adaptability to extreme environments, surviving and reproducing under conditions of prolonged dryness, pH levels ranging from 4.7 to 9.6, and temperatures from 0 to 45°C (1). Moreover, it exhibits high tolerance to elevated salt concentrations, enduring up to 30.5% salt at 4°C, making it prevalent in various environments (2). As a pathogenic bacterium, *Listeria monocytogenes* poses infections to humans, livestock, poultry, and wildlife. It can also parasitize ticks, flies, insects, fish, and crustaceans, and is widespread in river water, sludge, slaughterhouse waste, silage, dairy products, and seafood. Silage feed, in particular, is susceptible to contamination from soil and feces, promoting bacterial proliferation and contributing to a higher infection rate among ruminant animals such as cattle and sheep (1). In the 1970s, *Listeria monocytogenes* was identified as a pathogen responsible for human diseases, and in the 1980s, it was confirmed as a foodborne pathogen (3). Despite the relatively low number of infections each year (approximately 23,150 cases worldwide in 2010), human ingestion of contaminated food with *Listeria monocytogenes* remains the most common cause of infection. However, the mortality rate among infected individuals is notably high, ranging from 20 to 30%, surpassing that of other foodborne diseases (4). *Listeria monocytogenes* is a facultative intracellular parasite capable of traversing the host’s intestinal, blood–brain, and placental barriers. Upon breaching the intestinal barrier, it can induce bacteremia, septicemia, and febrile gastroenteritis. In humans, crossing the blood–brain barrier often results in meningitis and meningoencephalitis, while ruminant animals may develop encephalitis (5). Crossing the placental barrier may lead to miscarriage, stillbirth, or systemic fetal infection during the perinatal period (6–8). *Listeria monocytogenes* enters mammalian cells through surface proteins InlA and InlB-mediated internalization (9). InlA interacts with the calcium-dependent cell surface adhesion molecule E-cadherin (10), while InlB binds to the receptor for hepatocyte growth factor (HGF), known as tyrosine kinase c-Met (11). Host cells engulf *Listeria monocytogenes* via phagocytosis, forming phagosomes that can fuse with lysosomes to eliminate the pathogen. However, *Listeria monocytogenes* disrupts host cell defense mechanisms using various virulence factors, notably Listeriolysin O (LLO) (12). LLO disrupts phagosomal membranes, possesses pro-inflammatory properties, and accelerates tissue damage, facilitating immune evasion and bacterial proliferation (13). Following host cell infection, *Listeria monocytogenes* can induce multiple forms of cell death, including non-programmed cell death like necrosis, as well as programmed cell death such as autophagy, apoptosis, and pyroptosis (14–16).

Recent research has shed light on the ability of *Listeria monocytogenes* to induce host cell autophagy through LLO, thereby avoiding bacterial elimination (17). Autophagy is a fundamental process in eukaryotic cells, involving the degradation of damaged organelles and molecular components through lysosomal activity (18). The PINK1 and Parkin axis represents a critical mechanism of mitochondrial autophagy, utilizing the ubiquitin system to target mitochondria for autophagic degradation (19). LC3B, an autophagy-related gene, exists in two forms: type I, constitutively expressed in the cytoplasm, and type II, generated during autophagy. Type I LC3B undergoes processing and modification to bind to phosphatidylethanolamine (PE) on the autophagosomal membrane, forming type II LC3B (20). Type II LC3B remains on the membrane of autophagosomes, serving as a marker for cellular autophagic activity. Autophagy is a selective process crucial for cell survival during starvation, removal of damaged mitochondria, and maintenance of intracellular homeostasis (21). Additionally, it plays a pivotal role in the host defense against pathogens, as invading bacteria can be targeted by autophagy, either contained within vesicles or confined within the cytoplasm. However, *Listeria monocytogenes* can subvert autophagic mechanisms to enhance its pathogenicity (22). Cell apoptosis, a regulated process controlled by specific genes, is essential for normal development and the elimination of damaged or surplus cells. It also plays a crucial role in the body’s defense against diseases (23). Nevertheless, *Listeria monocytogenes* can exploit host cell apoptosis to further its pathogenicity. It can induce apoptosis in host lymphocytes, suppressing the immune response and promoting bacterial proliferation (24). Conversely, *Listeria monocytogenes* can maintain host cell viability by modulating the activity of LLO and ActA proteins, thereby preventing host cell death and facilitating bacterial proliferation (25).

Our study aimed to investigate the mechanisms underlying *Listeria monocytogenes* infection in the goat uterus and its impact on cell death pathways in uterine tissue. We analyzed changes in the protein and mRNA levels of E-cadherin and c-Met in goat uterus to elucidate the invasion process of *Listeria monocytogenes* following intravenous injection through the jugular vein. Additionally, we examined the protein and mRNA levels of LC3B, PINK1/Parkin, and Bax/Bcl-2 in goat uterine tissue to explore the influence of *Listeria monocytogenes* infection on cell autophagy and apoptosis. These findings provide valuable insights into the mechanisms of *Listeria monocytogenes* infection in goats and its impact on uterine tissue. They lay the groundwork for the development of clinical strategies for preventing *Listeria monocytogenes* infection in goats and the potential development of relevant medications.

## 2 Materials and methods

### 2.1 Animal grouping and sample collection

In this experiment, six healthy female Ganxi goats, aged 2.5 years, were selected. The goats were randomly divided into two groups: an infected group and a control group. The infected group was injected through the jugular vein with *Listeria monocytogenes*, which was identified and preserved by the Beef and Mutton Disease Control Laboratory of Yichun College. Meanwhile, the control group was injected with an equal volume of 0.9% sodium chloride solution through the jugular vein. Both groups of goats were raised separately using the same management methods. After the infected group showed clinical symptoms, goats from both groups were euthanized via the carotid artery. Uterine tissues from all goats were collected aseptically, washed with PBS, and part of the tissue was fixed with 4% paraformaldehyde. The remaining tissues were immediately frozen in liquid nitrogen and then stored in a −80°C freezer.

### 2.2 Giemsa staining

The method of Giemsa staining was used to observe *Listeria monocytogenes* in goat uterine tissue of the experimental group. The Giemsa staining kit (HB8397) was purchased from Qingdao Haibo Biotechnology Co., Ltd. In short, fixed uterine tissue is embedded in paraffin, followed by deparaffinization and hydration. The sections are then stained with Hematoxylin staining solution for 10–15 min, rinsed three times with distilled water, and finally mounted with neutral resin.

### 2.3 TUNEL

Using the Terminal deoxynucleotidyl transferase dUTP Nick End Labeling (TUNEL) staining method to observe apoptotic cells in uterine tissue. The TUNEL kit was purchased from Roche, Switzerland. In short, uterine tissue sections from the experimental and control groups are taken and subjected to deparaffinization and hydration. TUNEL staining is then performed according to the instructions provided with the kit. Apoptotic cells appear brown-yellow under the microscope, while normal cells appear blue. The apoptotic cell rate is calculated based on the observation of uterine tissue under the microscope.

### 2.4 Immunohistochemistry

Using immunohistochemistry staining method to observe the expression areas of *Listeria monocytogenes* bacterial protein, E-cadherin, c-Met, LC3B, PINK1, Parkin, Bax, and Bcl-2 proteins in uterine tissue sections. The Listeria tropina Rabbit pAb (bs-4581R) was purchased from Bioss company, E-cadherin antibody (AF0131) and c-Met antibody (AF6128) were obtained from Affinity company, Bcl-2 antibody (NM-009741) and Bax antibody (BC-014175) were purchased from proteintech company, LC3B (ab229327) and PINK1 (ab137361) were acquired from abcam company, and Parkin (bs-23687R) was obtained from Beijing Boaosen Biotechnology Co., Ltd. The rabbit SP kit and DAB chromogenic solution were purchased from Beijing Zhongshan Jinqiao Biotechnology Co., Ltd. In short, uterine tissue sections were deparaffinized and hydrated, followed by blocking and incubation with diluted primary antibodies overnight at 4°C. After washing three times with PBS, the sections were processed according to the instructions of the rabbit SP kit, and finally stained with DAB chromogenic solution. After chromogenic staining, counterstaining with hematoxylin was performed, followed by mounting with neutral resin. Brown indicates positive staining, while light yellow indicates weak positive staining.

### 2.5 RT-qPCR

Total RNA extraction from frozen uterine tissue using Trizol Reagent (CA92008) purchased from Ambion. cDNA synthesis using RNA reverse transcription kit and HiScript^®^ II QRT SuperMix for qPCR (R222-01) purchased from Nanjing Novozymes Biological Technology Co., Ltd. Real-time PCR (qPCR) performed using ChamQ SYBR qPCR Master Mix (Q311) purchased from Nanjing Novozymes Biological Technology Co., Ltd. Following the manufacturer’s instructions. Specific qPCR reaction conditions: Initial denaturation at 95°C for 3 min; Denaturation at 95°C for 10 s, annealing at 68°C for 30 s, for 40 cycles. Relative expression levels of autophagy and apoptosis-related genes calculated using the 2^−ΔΔCT^ method with β-actin as the internal reference (Table 1).

**Table 1:** Information of RT-qPCR Primers.

Gene	Primer sequence	Accession number	Product length
*E-cadherin*	F: TCGGAGGAGAAGGGTGGTCAAAG	NM_001317185.2	121
	R: CTGGCTCAAGTCAAAGTCCTGGTC		
*c-Met*	F:TGTGTGCGATTGGAGGAATGCC	NM_001317185.2	132
	R: AAAGTCCCAGCCACAAACAGTCAG		
*LC3B*	F: AGAAGGCGCTTACAGCTCAATGC	XM_018061829.1	92
	R: ACTTCACAAATCGGAGTGGACACAC		
*PINK1*	F: TCATCCAGCGAAGCCATCTTTAGC	XM_018055041.1	108
	R: TCCCTTGGGTCTTCCGTGAGTG		
*Parkin*	F: GCATAACGTGTACGGACATCAGGAG	XM_018053444.1	86
	R: CAGGTGGAAGCAGTCTAAGCAGATC		
*Bax*	F: GGCCCTTTTGCTTCAGGGTT	XM_018062750.1	121
	R: CAGACACTCGCYCAGCTTCT		
*Bcl-2*	F: GAGTTCGGAGGGGTCATGTG	NM_001314213.1	152
	R: TACAGCTCCACAAAGGCGTC		
*β-Actin*	F: CTCTTCCAGCCTTCCTTCCT	NM_001314342.1	177
	R: GGGCAGTGATCTCTTTCTGC		

### 2.6 Western blot

Total protein extraction from uterine tissue using High-Efficiency RIPA Tissue/Cell Lysis Kit purchased from Solarbio. Determine protein concentration using the BCA protein quantification kit purchased from Beijing Pulei Gene Technology Co., Ltd. SDS-PAGE gel preparation using the SDS-PAGE Gel Preparation Kit (AR0138) purchased from BioDee Biology. Load protein samples onto the gel along with the SDS-PAGE Protein Loading Buffer (denaturing) (AR1112-10). Perform SDS-PAGE electrophoresis. Transfer the separated proteins from the gel to a PVDF membrane. Incubate the PVDF membrane in 5% skim milk for 2 h at room temperature. Incubate the PVDF membrane with Anti-Listeriolysin antibody (1:1,000), E-cadherin antibody (1:1,000), c-Met antibody (1:1,000), LC3B antibody (1:1000), PINK1 antibody (1:1000), Parkin antibody (1:1,000), Bax antibody (1:1,000), and Bcl-2 antibody (1:1,000) overnight at 4°C. Wash the PVDF membrane with TBST buffer. Incubate the PVDF membrane with Goat Anti-Rabbit IgG secondary antibody (ab6721) diluted at 1:1,0000 for 2 h at room temperature. Wash the PVDF membrane with TBST buffer. Perform chemiluminescent detection using the Ultra-sensitive Chemiluminescence Detection Kit (GS0720) purchased from US EVERBRIGHT INC.

### 2.7 Data analysis

Statistical Analysis Using GraphPad Prism 8.0. Perform a *t*-test to analyze the differences between the two groups. Consider a *p*-value less than 0.05 (*p* < 0.05) as statistically significant.

## 3 Results

### 3.1 The distribution and expression of *Listeria monocytogenes* and LLO proteins in the goat uterus

**Figure 1:**
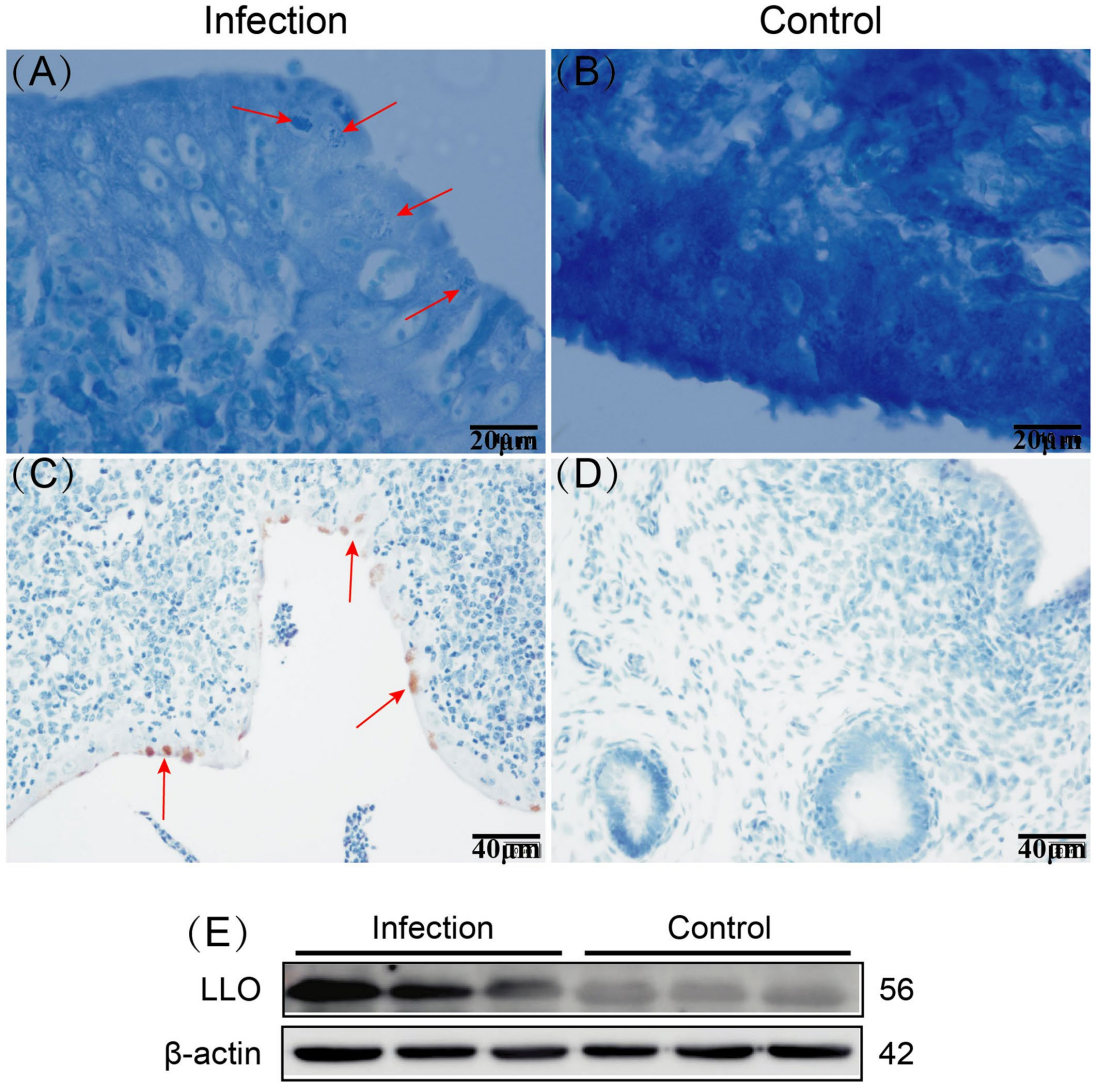
Giemsa staining results of infected and control goat uterine tissue, with red arrows indicating bacteria **(A,B)**. Immunohistochemistry staining results of infected and control goat uterine tissue, with red arrows indicating positive areas **(C,D)**. Expression of LLO protein in infected and control goat uterine tissue **(E)**.

The Giemsa staining results revealed the presence of bacteria within the epithelial cells of the infected goat uterine tissue, appearing clustered and stained dark blue (Figure 1A). Immunohistochemistry staining showed brown positive areas within the epithelial cells of the uterine mucosa (Figure 1C), consistent with the location of bacteria observed in the Giemsa staining results. Western blot analysis demonstrated that LLO protein was only expressed in the infected goat uterine tissue and not in the control group (Figure 1E). These findings indicate successful invasion of *Listeria monocytogenes* into the uterine tissue of goats following intravenous injection, with colonization observed within the epithelial cells of the uterine mucosa.

### 3.2 The localization and expression of E-cadherin and c-Met in the goat uterus

**Figure 2:**
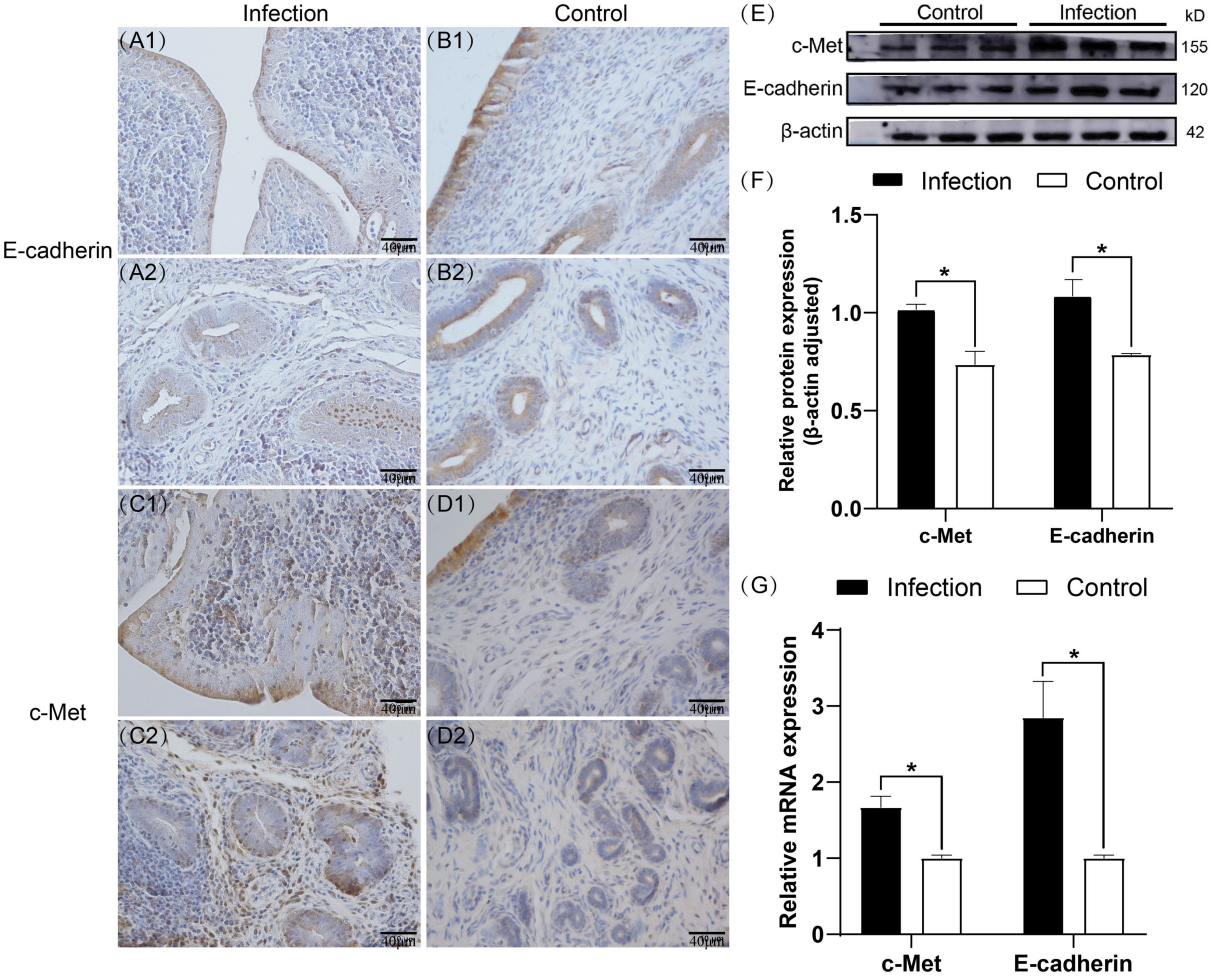
Expression patterns of E-cadherin and c-Met proteins in uterine tissues **(A1–D2)**. Expression levels of E-cadherin and c-Met proteins in uterine tissues **(E,F)**. Expression levels of E-cadherin and c-Met mRNA in uterine tissues **(G)**. The brown area indicates the location of the target protein.*p < 0.05 vs. Control.

Immunohistochemistry staining results demonstrated that both E-cadherin and c-Met were expressed in the goat uterus of both the infection group and the control group (Figures 2A1–D2), and they were localized to the same area. E-cadherin exhibited positive expression in the endometrial epithelial cells and uterine glandular epithelial cells, while other cells did not express it. In the control group, c-Met showed strong positive expression in the endometrial epithelial cells and weak expression in the uterine glandular epithelial cells. However, in the infection group, c-Met not only exhibited strong positive expression in the endometrial epithelial cells and uterine glandular epithelial cells but also in some cells in the lamina propria. Western blot results demonstrated that compared to the control group, the relative expression levels of E-cadherin and c-Met proteins in the uterus of the infection group were significantly increased (*p* < 0.05) (Figures 2E,F). RT-qPCR results indicated that compared to the control group, the mRNA expression levels of E-cadherin and c-Met were significantly elevated in the infection group (*p* < 0.05) (Figure 2G).

### 3.3 The localization and expression of LC3B, PINK1, and Parkin in the goat uterus

**Figure 3:**
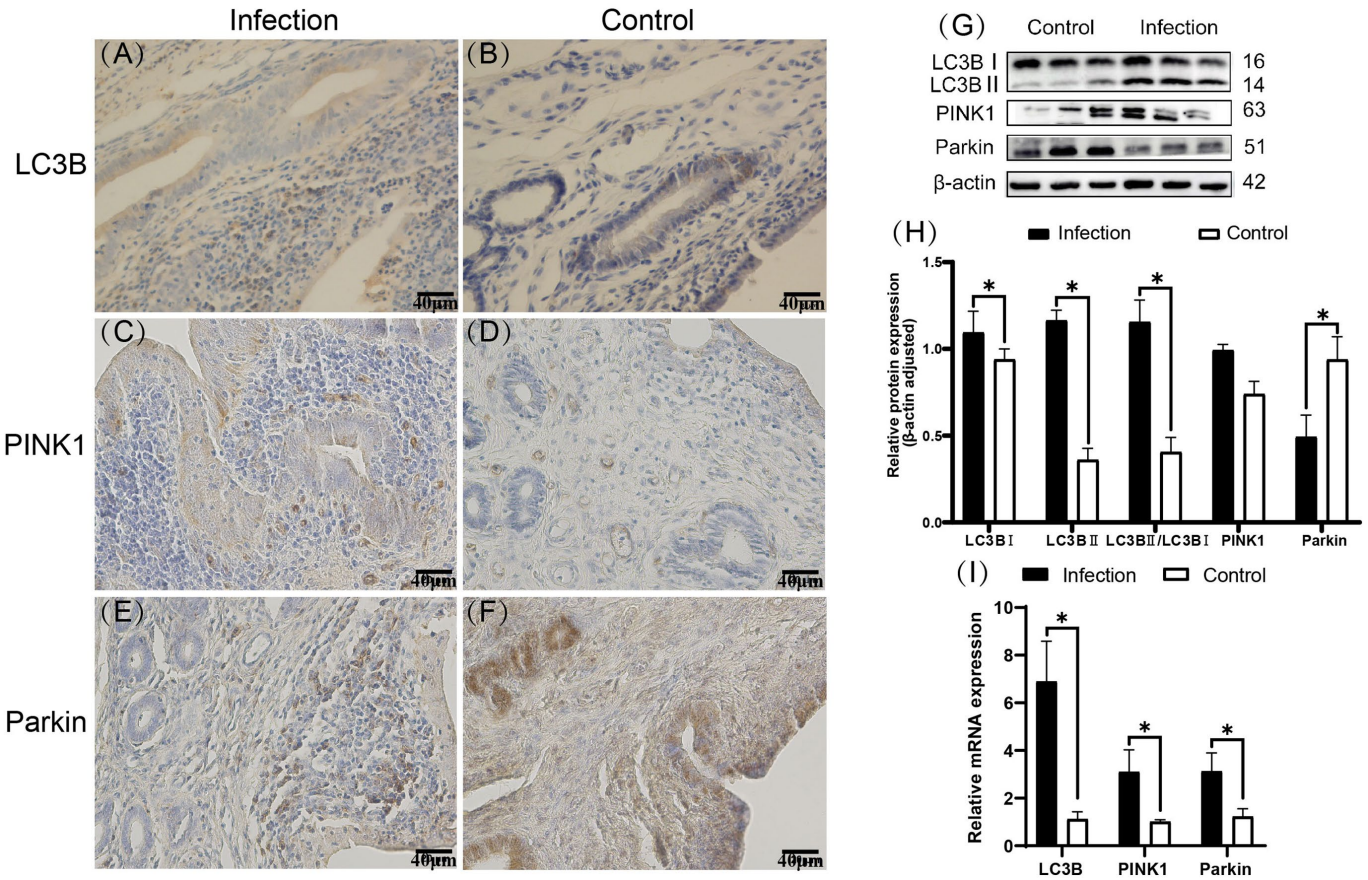
Expression patterns of LC3B, PINK1, and Parkin proteins in uterine tissues **(A–F)**. Expression levels of E-cadherin and c-Met proteins in uterine tissues **(G,H)**. Expression levels of E-cadherin and c-Met mRNA in uterine tissues **(I)**. The brown area indicates the location of the target protein. *p < 0.05 vs. Control.

Immunohistochemistry staining results revealed that LC3B, PINK1 and Parkin proteins were expressed in the goat uterus in both the infected and control groups (Figures 3A–F), displaying similar expression patterns. LC3B was mainly expressed in the uterine mucosal and submucosal layers, with positive expression observed in uterine epithelial cells, stromal cells, and uterine gland cells. PINK1 was mainly expressed in the uterine mucosal and submucosal layers. Unlike LC3B, PINK1 protein was also expressed in the endothelial cells of the stromal layer blood vessels. The regions of Parkin protein expression ware the same as those of LC3B protein expression. The Western blot results (Figures 3G,H) indicated that compared to the control group, the expression levels of LC3BII and PINK1 proteins, as well as the ratio of LC3BII/LC3BI, significantly increased in the infected group (*p* < 0.05). Conversely, the expression level of Parkin protein significantly decreased (p < 0.05). The RT-qPCR results (Figure 3I) showed that compared to the control group, the mRNA expression levels of LC3B, PINK1 and Parkin genes in the uteri of the infected group significantly increased (*p* < 0.05).

### 3.4 The localization and expression of apoptotic cells and Bax/Bcl-2 in the goat uterus

**Figure 4:**
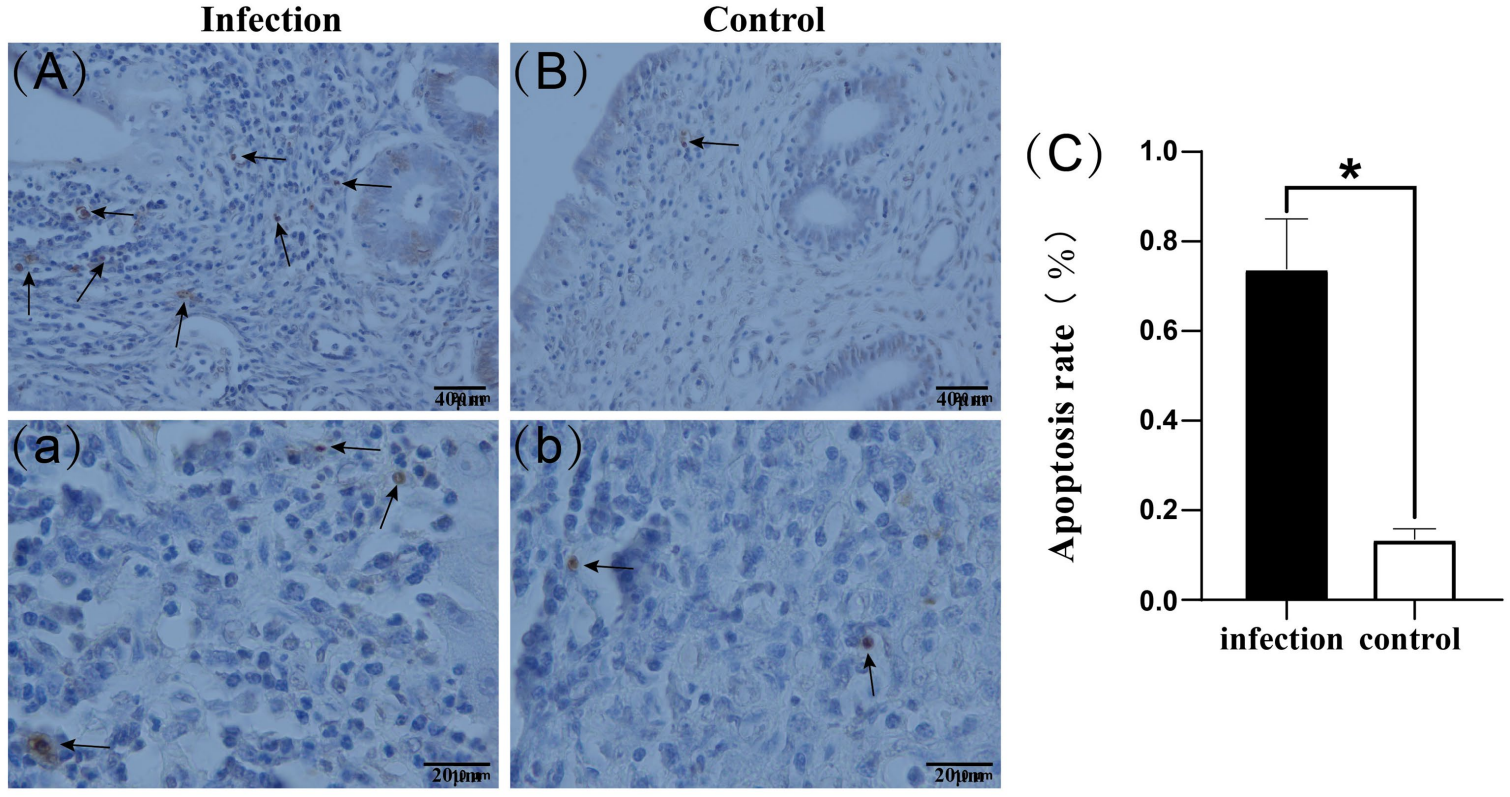
Apoptotic cells in uterine tissue **(A–b)**. Apoptotic cell rate **(C)**. Black arrows indicate apoptotic cells.*p < 0.05 vs. Control.

Our findings revealed that apoptotic cells were primarily located in the stroma, with some also observed in the uterine glands. Furthermore, the number of apoptotic cells in the uteri of the infected group surpassed that of the control group (Figures 4A–b). Apoptotic cell rates were determined by counting the apoptotic cells in each field of view, revealing that the apoptotic cell rate in the infected group was significantly higher than that in the control group (*p* < 0.05) (Figure 4C).

**Figure 5:**
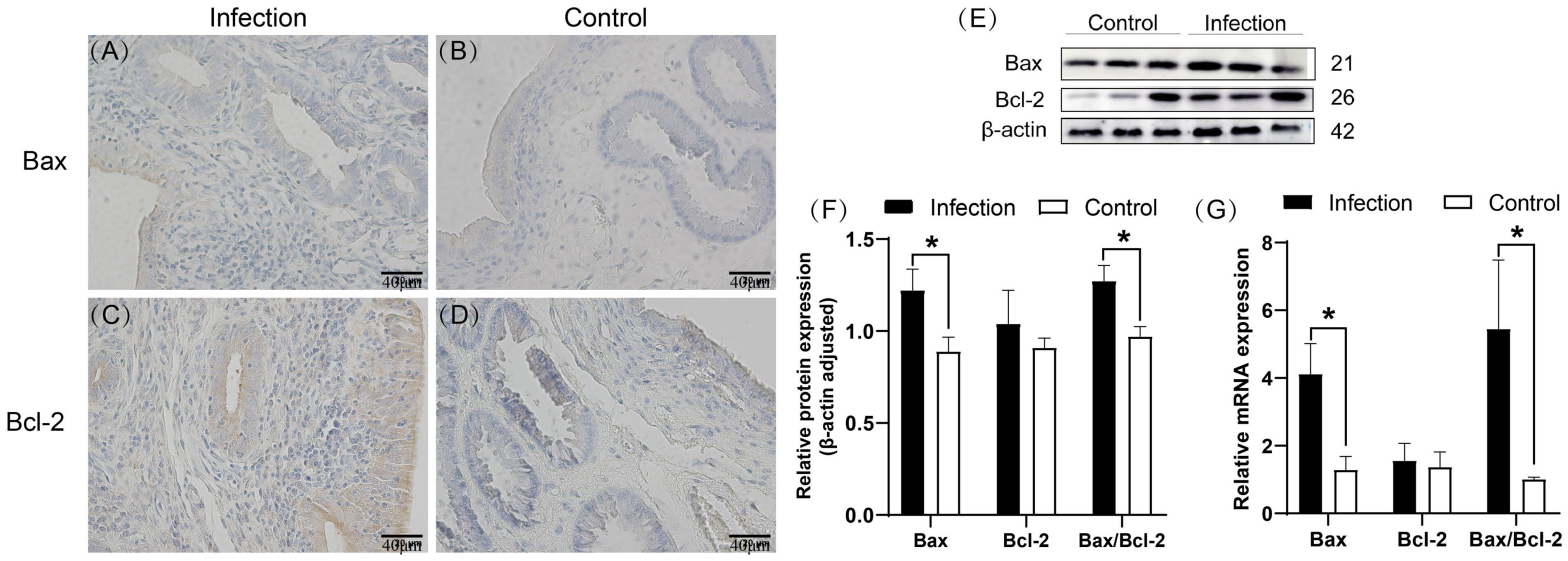
Expression patterns of Bax and Bcl-2 proteins in uterine tissue **(A–D)**. Levels of Bax and Bcl-2 proteins in uterine tissue **(E,F)**. mRNA levels of Bax and Bcl-2 genes in uterine tissue **(G)**. The brown area indicates the location of the target protein. *p < 0.05 vs. Control.

Immunohistochemical staining results revealed predominant expression of Bax in the uterine mucosal epithelial cells, whereas Bcl-2 was found in both the mucosal and submucosal layers, with positive areas also detected in uterine glands (Figures 5A–D). Western blot analysis demonstrated a significantly higher expression level of Bax protein in the infected group uterus compared to the control group (*p* < 0.05), while the expression level of Bcl-2 protein was also elevated in the infected group, although without statistical significance (*p* > 0.05). Notably, the Bax/Bcl-2 ratio was significantly higher in the infected group than in the control group (*p* < 0.05) (Figures 5E,F). qRT-PCR results showed that in the infected group uterus, the mRNA levels of the Bax gene and the Bax/Bcl-2 ratio were significantly increased compared to the control group (*p* < 0.05), whereas there was no significant difference in the mRNA expression level of the Bcl-2 gene (*p* > 0.05) (Figure 5G).

## 4 Discussion

Since its discovery in the last century, *Listeria monocytogenes* (LM) has remained a persistent threat to the health of humans and livestock, presenting significant challenges to the animal husbandry and animal food processing industries. LM exhibits the ability to traverse various barriers within its hosts, including the intestinal, placental and blood–brain barriers (26). Infection with LM can result in bacterial septicemia and meningitis in animals with compromised immune systems, and it can lead to miscarriages in pregnant animals, among other clinical manifestations (27). The bacterium enters intestinal epithelial cells through multiple pathways and subsequently disseminates to target organs via the lymphatic and circulatory systems, facilitating invasion into various tissues and organs such as the brain and placenta (28). LM employs a range of virulence factors and complex mechanisms to enhance bacterial survival within host cells during infection, including InlA, InlB, ActA, LLO, PI-PLC, and PC-PLC (29). Of these, LLO plays a pivotal role in the pathogenic process, making it one of LM’s most significant virulence factors. Confirmation of bacterial presence in the epithelial cells of goat uterine mucosa in the infected group was achieved through Gram staining. Immunohistochemistry further identified these bacteria as LM. Additionally, using the Western blot method, we assessed the expression of Listeriolysin (LLO) in the uteri of both control and infected goat groups, revealing LLO protein expression solely in the infected group. These findings unequivocally establish the presence of LM in the uteri of infected goats and demonstrate the expression of LLO by LM to facilitate bacterial infection of the goat uterus. This serves as a critical foundation for further investigation into the mechanisms of LM infection in goat uterine cells and the impact of infection on uterine cell autophagy and apoptosis.

In ruminants infected with *Listeria monocytogenes* (LM), miscarriage is a prevalent occurrence, often observed in the later stages of pregnancy. This can result in fetal death and the development of endometritis in the mother (30). E-cadherin, a transmembrane glycoprotein, plays a pivotal role in maintaining tissue stability by mediating adhesive connections within the polarized tissue of the placenta. While its extracellular domain typically engages in homotypic interactions, the cytoplasmic domain interacts with the actin cytoskeleton. LM disrupts the physiological function of E-cadherin through its internalin protein, InlA, promoting cortical actin polymerization and membrane rearrangement, facilitating cellular invasion and crossing of the fetal-maternal barrier (31, 32). In the placenta, InlB collaborates with InlA to facilitate cellular invasion, allowing LM to enter non-polarized epithelial cells *in vitro* and aiding placental invasion *in vivo* (33, 34). Research on LM’s traversal of the ruminant placental barrier has advanced, but the mechanisms of LM’s colonization in the ruminant uterus post-transcendence remain less understood. The binding of InlA to E-cadherin and InlB to c-Met is crucial for LM’s passage through the placental barrier (35). Thus, it is speculated whether InlA and InlB also play roles in LM invading the goat uterus. By studying changes in E-cadherin and c-Met within the host, we analyzed whether LM utilizes the InlA and InlB pathways during uterine invasion. Immunohistochemistry results demonstrated that both the infected and control groups expressed E-cadherin and c-Met proteins in the endometrial epithelial cells and glandular epithelial cells, with the infected group showing higher expression intensity. Additionally, some cells in the intrinsic layer of the uterus in the infected group expressed c-Met protein. RT-qPCR experiments revealed a significant increase in the mRNA expression levels of E-cadherin and c-Met genes in the infected group compared to the control group (*p* < 0.05). Western blot further confirmed the expression changes of E-cadherin and c-Met proteins, indicating a significant increase in the infected group compared to the control group (*p* < 0.05). In summary, our study indicates that the expression levels of host E-cadherin and c-Met genes and proteins significantly increase during LM’s invasion of the goat uterus. Further studies on the expression of Listeria internalin proteins InlA and InlB are needed to confirm their involvement in LM’s invasion of the goat uterus. However, existing research suggests that InlA promotes bacterial internalization and adhesion to the host cell surface through interaction with E-cadherin, while InlB interacts with c-Met, leading to actin cytoskeleton rearrangement and bacterial uptake. These findings highlight the complex mechanisms underlying LM infection in ruminants (36–38).

Recent research indicates that *Listeria monocytogenes* (LM) can evade cellular killing by inducing autophagy in host cells through the action of the virulence factor listeriolysin O (LLO) (20). LLO mediates the oligomerization of the mitochondrial autophagy receptor NLRX1, facilitating its interaction with LC3B in the LC3B interaction region, inducing mitochondrial autophagy (39). This process eliminates bactericidal substances produced by mitochondria, thus mitigating their impact on the survival of LM (40). Our experimental findings, supported by immunohistochemistry, reveal the expression of LC3B, PINK1 and Parkin proteins in both infected and control groups within the uterine tissue of goats. LC3B expression was primarily observed in the uterine mucosa and submucosa, including uterine epithelial cells, stromal cells and glandular cells. Similar to LC3B, Parkin was mainly expressed in these areas. However, PINK1 was positively expressed in the endothelial cells within the stroma, differing from LC3B and Parkin, suggesting that *Listeria monocytogenes* infection may lead to differential regulation of autophagy-related proteins across various cell types. RT-qPCR results showed a significant increase in mRNA expression levels of LC3B, PINK1 and Parkin genes in the uteri of the infected group compared to the control group (*p* < 0.05), indicating significant changes in the expression of autophagy-related genes in uterine cells due to LM infection. Western blot analysis further confirmed that, compared to the control group, the expression of LC3B-II protein and the ratio of LC3B-II/LC3B-I were significantly increased in the infected group(*p* < 0.05), while the expression of Parkin protein was significantly decreased(*p* < 0.05).

These findings suggest that LM infection may regulate the expression levels of autophagy-related proteins in different ways. The increase in LC3B and the LC3BII/LC3BI ratio likely indicates heightened autophagic activity, whereas the opposing regulation of PINK1 and Parkin suggests that the control of autophagy by *Listeria monocytogenes* infection is not unidirectional. The PINK1/Parkin pathway-mediated mitochondrial autophagy plays a crucial role in maintaining mitochondrial quality and homeostasis, participating in the cellular antiviral immune response, promoting pathogen clearance, enhancing innate immune responses, and protecting immune cell survival. Blocking mitochondrial autophagy leads to the accumulation of damaged mitochondria, increasing the production of mitochondrial reactive oxygen species (mROS), ultimately inhibiting the proliferation of LM (17).

After infection with *Listeria monocytogenes*, the host immune response plays a crucial role in limiting these infections. In the early stages of infection, the host primarily clears *Listeria monocytogenes* through inflammation-mediated nonspecific immune responses (41). Because *Listeria monocytogenes* is an intracellular parasite, in the later stages of infection, the host needs to rely on lymphocyte-mediated specific cell-mediated immunity to combat *Listeria monocytogenes* infection (42). Through TUNEL apoptosis detection, we observed a significant increase in the number and rate of apoptotic cells in the goat uterus after infection with *Listeria monocytogenes*. This indicates that bacterial infection induces apoptosis of uterine cells, suggesting its potential impact on uterine tissue homeostasis. Previous studies have shown that *Listeria monocytogenes* can suppress host immune responses by promoting lymphocyte apoptosis (24), which is consistent with our experimental results. Transcription and expression of the Bax and Bcl-2 genes are major influencing factors in the mitochondrial pathway-mediated apoptosis. Bax induces changes in mitochondrial membrane structure, leading to the release of cytochrome c from the mitochondria into the cytoplasm. Cytochrome c then activates the protein Apaf-1, which, along with activated Apaf-1, initiates the formation of apoptotic bodies by binding to caspase-9, an apoptotic protease, thereby activating downstream effector caspases and initiating apoptosis. Conversely, Bcl-2 exerts the opposite effect by inhibiting Bax, thereby suppressing apoptosis (43). Cell apoptosis, especially apoptosis of infected cells, is another immune response by the host to clear pathogenic microorganisms (44). At the genetic level, we found that compared to the control group, the Bax gene and Bax/Bcl-2 ratio was significantly upregulated in the infected group, while the expression of the Bcl-2 gene showed no significant change. This is consistent with the results of Western blot analysis, where Bax protein expression increased significantly while Bcl-2 protein expression showed no significant difference. These findings suggest that infection may lead to changes in the balance of apoptosis regulation, favoring a state that promotes cell apoptosis. The results of immunohistochemistry showed that after infection with *Listeria monocytogenes*, the expression of Bax and Bcl-2 proteins in the uterus was upregulated, but their distribution and location did not change significantly. This suggests that these proteins may play more complex regulatory roles under infection conditions and participate in multiple biological processes within the cell.

## 5 Conclusion

In summary, our study revealed the following key points: Firstly, injection of *Listeria monocytogenes* into goats resulted in Listeria infection in the goat uterus. Secondly, during the infection process, interaction between *Listeria monocytogenes* and goat uterine cells was observed, leading to a significant increase in the expression levels of Listeriolysin O (LLO) and the host uterine tissue proteins E-cadherin and c-Met. This suggests that *Listeria monocytogenes* may infect the goat uterus via two pathways: InlA/E-cadherin and InlB/c-Met. However, further research is needed to investigate the expression of the internalization proteins InlA and InlB of *Listeria monocytogenes* in goat uterine tissue to gain a deeper understanding of the molecular mechanisms involved in this process. *Listeria monocytogenes* infection can trigger cell apoptosis and autophagy in the goat uterus. The expression and localization of apoptosis-related genes Bcl-2 and Bax, as well as autophagy-related genes LC3B, PINK1 and Parkin, show varying degrees of alteration in the goat uterus, influencing the occurrence of cell apoptosis and autophagic responses. Our research underscores the diverse impacts of *Listeria monocytogenes* infection on goat uterine cells, resulting in increased apoptosis and elevated levels of autophagy. However, achieving a comprehensive understanding of these regulatory mechanisms remains a challenging task. Moving forward, we will delve deeper into exploring the potential consequences of *Listeria monocytogenes* infection on uterine health and investigating potential therapeutic approaches.

## Data Availability

The original contributions presented in the study are included in the article/Supplementary material, further inquiries can be directed to the corresponding authors.
